# Clinical and Pharmacotherapeutic Profile of Patients with Type 2 Diabetes Mellitus Admitted to a Hospital Emergency Department

**DOI:** 10.3390/biomedicines11020256

**Published:** 2023-01-18

**Authors:** António Cabral Lopes, Olga Lourenço, Fátima Roque, Manuel Morgado

**Affiliations:** 1 Pharmaceutical Services of Unity Local of Health of Guarda (ULS da Guarda), 6300-035 Guarda, Portugal; 2 Health Sciences Faculty, University of Beira Interior (FCS-UBI), 6200-506 Covilhã, Portugal; 3 Health Sciences Research Centre, University of Beira Interior (CICS-UBI), 6200-506 Covilhã, Portugal; 4 Research Unit for Inland Development, Polytechnic Institute of Guarda (UDI-IPG), 6300-559 Guarda, Portugal; 5 Pharmaceutical Services of University Hospital Center of Cova da Beira, 6200-251 Covilhã, Portugal

**Keywords:** Type 2 diabetes mellitus, glycemia, cardiovascular disorders, kidney function

## Abstract

Type 2 diabetes mellitus (T2DM) is closely associated with other pathologies, which may require complex therapeutic approaches. We aim to characterize the clinical and pharmacological profile of T2DM patients admitted to an emergency department. Patients aged ≥65 years and who were already using at least one antidiabetic drug were included in this analysis. Blood glycemia, creatinine, aspartate aminotransferase (AST), alanine aminotransferase (ALT), and hemoglobin were analyzed for each patient, as well as personal pathological history, diagnosis(s) at admission, and antidiabetic drugs used before. Outcome variables were analyzed using Pearson’s Chi-Square, Fisher’s exact test, and linear regression test. In total, 420 patients were randomly selected (48.6% male and 51.4% female). Patients with family support showed a lower incidence of high glycemia at admission (*p* = 0.016). Higher blood creatinine levels were associated with higher blood glycemia (*p* = 0.005), and hyperuricemia (HU) (*p* = 0.001), as well as HU, was associated with a higher incidence of acute cardiovascular diseases (ACD) (*p* = 0.007). Hemoglobin levels are lower with age (*p* = 0.0001), creatinine (*p* = 0.009), and female gender (*p* = 0.03). The lower the AST/ALT ratio, the higher the glycemia at admission (*p* < 0.0001). Obese patients with (*p* = 0.021) or without (*p* = 0.027) concomitant dyslipidemia had a higher incidence of ACD. Insulin (*p* = 0.003) and glucagon-like peptide-1 agonists (GLP1 RA) (*p* = 0.023) were associated with a higher incidence of decompensated heart failure, while sulfonylureas (*p* = 0.009), metformin-associated with dipeptidyl peptidase-4 inhibitors (DPP4i) (*p* = 0.029) or to a sulfonylurea (*p* = 0.003) with a lower incidence. Metformin, in monotherapy or associated with DPP4i, was associated with a lower incidence of acute kidney injury (*p* = 0.017) or acute chronic kidney injury (*p* = 0.014). SGLT2i monotherapy (*p* = 0.0003), associated with metformin (*p* = 0.026) or with DPP4i (*p* = 0.007), as well as insulin and sulfonylurea association (*p* = 0.026), were associated with hydroelectrolytic disorders, unlike GLP1 RA (*p* = 0.017), DPP4i associated with insulin (*p* = 0.034) or with a GLP1 RA (*p* = 0.003). Insulin was mainly used by autonomous and institutionalized patients (*p* = 0.0008), while metformin (*p* = 0.003) and GLP1 RA (*p* < 0.0001) were used by autonomous patients. Sulfonylureas were mostly used by male patients (*p* = 0.027), while SGLT2 (*p* = 0.0004) and GLP1 RA (*p* < 0.0001) were mostly used by patients within the age group 65–85 years. Sulfonylureas (*p* = 0.008), insulin associated with metformin (*p* = 0.040) or with a sulfonylurea (*p* = 0.048), as well as DPP4i and sulfonylurea association (*p* = 0.031), were associated with higher blood glycemia. T2DM patients are characterized by great heterogeneity from a clinical point of view presenting with several associated comorbidities, so the pharmacotherapeutic approach must consider all aspects that may affect disease progression.

## 1. Introduction

Diabetes mellitus (DM) is a common condition affecting about 483 million people worldwide and the total number of adults (20–79 years) with the disease in 2045 is estimated to increase to 629 million [1]. If left untreated, DM can cause other life-threatening conditions such as cardiovascular diseases, stroke, chronic kidney disease (CKD), foot ulcers, eye damage, and neuropathy. To date, there is no permanent cure for DM, and patients need to ensure a healthy lifestyle and comply with the pharmacological therapy that best suits their condition [2]. Patients with DM, especially those who are older (≥65), have a lower socioeconomic status, lower health literacy, and do not have familiar or institutional support, are often admitted to hospital emergency services either due to poor glycemic control or to the lack of control of other comorbidities that are closely related. Health professionals must coordinate their intervention to improve glycemic control while maintaining safety, contributing to the improvement of long-term clinical outcomes, and ensuring the sustainability of national healthcare systems [3,4,5].

Type II diabetes mellitus (T2DM) is the most prevalent form of DM (90%), and it is mainly caused by the combination of two factors: peripheral tissue resistance to insulin and insufficient insulin secretion by pancreatic β cells [6,7]. The increase in the prevalence of T2DM has been mainly driven by rapid urbanization, changes in lifestyle, and poor dietary patterns [8,9]. Several risk factors have been identified for T2DM, such as hyperuricemia, sleep disorders, smoking, depression, cardiovascular disease, dyslipidemia, high blood pressure, aging, ethnicity, family history, physical inactivity, and obesity [10,11,12,13,14,15].

The provision of health care in this area has evolved over the last few decades, either in the growing specialization of health professionals, increasingly integrated into multidisciplinary teams, or in the emergence of new antidiabetic drugs. These new drugs have allowed not only better glycemic control but also demonstrated protective effects in other concomitant pathologies that greatly contribute to the increase in morbidity and mortality rates in T2DM [16,17]. On the other hand, antidiabetic drugs are increasingly being studied due to the adverse effects that may result from their use, namely their impact on cardiorenal function and electrolytic balance [18,19]. Sodium-glucose co-transporter-2 inhibitors (SGLT2i), Dipeptidyl peptidase-4 inhibitors (DPP4i), and Glucagon-like peptide-1 agonists (GLP1 AR), in addition to hypoglycemic effect, have demonstrated cardiorenal protective effects, while SGLTi has been associated with hydroelectrolytic disorders [20,21].

Insulin, metformin, and sulfonylureas are some of the classic drug classes used to treat T2DM. More recently, new therapeutic approaches have emerged, such as SGLT2i, which increases urinary glucose elimination and blocks its renal absorption; GLP1 AR, which increases insulin secretion by reducing glucagon secretion, delays gastric emptying and promotes satiety; and DPP4i, which inhibits incretin degradation promoting increased insulin secretion and reduced glucagon secretions [22,23,24]. 

This work aims to characterize the clinical and pharmacological profile of patients with T2DM who were admitted to the emergency department of the Local Health Unit of Guarda (LHUG), identifying how their clinical condition, on admission time, can be influenced by pre-existing comorbidities and by antidiabetic drugs previously used.

## 2. Materials and Methods

### 2.1. Study Design and Sample

A retrospective study of 420 patients with T2DM admitted to the LHUG emergency department from June 2019 to September 2022 was performed. Ethics Committee of the LHUG, Emergency, and Internal Medicine Departments Director’s authorizations were obtained.

About 140 patients are admitted to the LHUG emergency department per day, of which about 60% have a known diagnosis of T2DM. Our study includes 39 months of analysis, which amounts to approximately 163,800 admissions episodes in that period (approximately 98,280 patients with a previously known diagnosis of T2DM). This means 383 or more measurements are needed to have a confidence level of 95% that the real value is within ±5% of the measured value.

**Figure 1:**
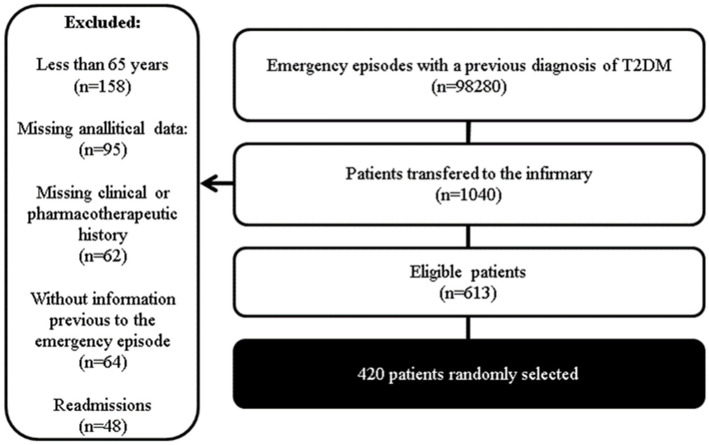
Sample selection.

Patients with the following characteristics were randomly selected (Figure 1):

✓ Diagnosis of T2DM before admission to the emergency department;
✓ At least 65 years of age;
✓ At least one antidiabetic drug included in the chronic treatment plan before admission;
✓ Complete and objective information described in the clinical diary regarding chronic medication, clinical history, and reason(s) for admission to the Emergency Department;
✓ Complete information regarding the analytical parameters included in the study upon admission;
✓ After applying these criteria, a sample of 613 patients was obtained, from which 420 were randomly selected.

### 2.2. Data Collection

Data on patients with T2DM were obtained from the SClinico^®^ and Modulab^®^ platforms. The following variables were analyzed:

✓ Age, gender, and condition (autonomous, family support, or institutionalized);
✓ Bioanalytical parameters at admission: blood glycemia; creatinine; hemoglobin; aspartate aminotransferase (AST), and alanine aminotransferase (ALT);
✓ Personal pathological history: T2DM; high blood pressure (HBP); heart failure (HF); atrial fibrillation (AF); acute myocardial infarction (AMI); dyslipidemia; chronic kidney disease (CKD); hyperuricemia (HU); stroke, obesity; chronic liver disease (CLD); oncological disease (OD); chronic obstructive pulmonary disease (COPD); alcoholism; chronic anemia (CA);
✓ Diagnosis that justifies admission: decompensated heart failure (DHF); acute chronic kidney disease (ACKD); acute kidney injury (AKI); urinary tract infection (UTI); pulmonary embolism (PE); stroke; AMI; respiratory tract infection (RTI); hydroelectrolytic disorders (HED); bleeding; gastroenteritis; acute chronic liver disease (ACLD); pancreatitis; hypoglycemia; respiratory failure; sepsis;
✓ Antidiabetic drugs included in the therapeutic plan before admission: insulin; SGLT2i; DPP4i; GLP1 RA; metformin and sulfonylureas.

### 2.3. Statistical Analysis

For the statistical analysis, IBM SPSS statistics 28 (IBM, Armonk, NY, USA) was used. Categorical variables were described through their respective absolute and relative frequencies (percentages). Pearson’s Chi-Square, Fisher’s exact test, and linear regression tests were used with a statistical significance level of 5% (*p* < 0.05).

## 3. Results

### 3.1. Sample Characterization

The variables were analyzed in order to verify possible relationships between patients’ characteristics (condition, gender, age, laboratory parameters, pathological and pharmacological history) admitted to the emergency department and diagnosis at admission (Table 1).

The American Diabetes Association classifies glycemia above 180 mg/dL as hyperglycemia; therefore, patients were classified according to this reference value [25].

A cut-off value of 1.2 mg/dL was considered for blood creatinine (for men with normal kidney function is approximately 0.6 to 1.2mg/dL and between 0.5 to 1.1 mg/dL for women) [26].

### 3.2. Relationship between Patient’s Condition before Admission, Age, and Gender with Glycemia Levels

There was a statistically significant association between the patient’s condition and glycemia levels at admission (*p* = 0.016, Pearson’s Chi-Square). Patients with family support showed a lower incidence of hyperglycemia (9.5%) than autonomous (23.1%) and institutionalized (23.8%) (Table 2). On the other hand, there was no statistically significant association between gender and glycemia levels (*p* = 0.862, Pearson’s Chi-Square), as well as between age and glycemia levels at admission (*p* = 0.281, Pearson’s Chi-Square).

### 3.3. Hyperuricemia, Hemoglobin, and AST/ALT Ratio

#### 3.3.1. Hyperuricemia

According to our results, there was a statistically significant association between blood creatinine levels and glycemia (*p* = 0.005, Pearson’s Chi-Square) and HU (*p* = 0.001, Pearson’s Chi-Square). Patients with blood glycemia levels above 180 mg/dL and a history of HU had higher blood creatinine values (≥1.2 mg/dL) at admission. There was also a statistically significant association between HU and acute CVD (decompensated heart failure, stroke, acute myocardial infarction, and/or pulmonary embolism) at admission (*p* = 0.007, Pearson’s Chi-Square).

#### 3.3.2. Hemoglobin and T2DM

In order to relate hemoglobin levels at admission to gender, age, and blood creatinine (<1.2 mg/dL or ≥1.2 mg/dL), we used a linear regression model using the (“stepwise” method). It was observed that the variables age (*p* = 0.0001), creatinine (*p* = 0.001), and gender (*p* = 0.03) were significant in the model. The regression equation obtained (Hb = 17.130–0.056 (Age)—0.766 (Creatinine above 1.2 mg/dL)—0.492 (Gender)) indicates that hemoglobin values decrease with age, being lower in female patients and with creatinine values above 1.2 mg/dL.

#### 3.3.3. AST/ALT Ratio

The relationship between glycemia (converted to the Neperian logarithm) and the AST/ALT ratio was analyzed through a linear regression model. According to the regression equation (Ln(Glycemia)) = 5.503–0.159 (AST/ALT ratio), the lower the AST/ALT ratio, the higher the glycemia levels at admission (*p* < 0.0001).

### 3.4. Obesity, Dyslipidemia, and Acute Cardiovascular Disorders

Obese patients with or without concomitant dyslipidemia (Table 1) were exposed to a higher risk of acute cardiovascular disorders (DHF, AMI, Stroke, and/or PE) at admission (OR 2.049, 95% CI 1.075–3.906, *p* = 0.027, Pearson’s Chi-Square) and (OR 1.825, 95% CI 1.092–3.051, *p* = 0.021, Pearson’s Chi-Square), respectively than non-obese patients.

### 3.5. Antidiabetic Therapy and Heart Failure

Our findings suggest that patients using insulin (*p* = 0.003), GLP1 RA (*p* = 0.023) or both associated (*p* = 0.007) were exposed to a higher risk of DHF at admission, while patients using sulfonylureas (*p* = 0.009), metformin and DPP4i association (*p* = 0.029) and metformin and sulfonylurea association (*p* = 0.003) to a lower risk (Table 3).

### 3.6. Antidiabetic Therapy and Kidney Function

Patients undergoing treatment with metformin (*p* = 0.017) or metformin and DPP4i association (*p* = 0.014) were exposed to a lower risk of AKI or ACKD (Table 4).

Patients using SGLT2i (*p* = 0.0003), insulin and sulfonylurea association (*p* = 0.026), metformin and SGLT2i association (OR *p* = 0.026), and DPP4i and SGLT2i association (*p* = 0.007) were exposed to a higher risk of hydroelectrolytic disorders. On the other hand, patients using GLP1 RA (*p* = 0.017), insulin and DPP4i association (*p* = 0.034), or DPP4i and GLP1 RA association (*p* = 0.003) had a lower risk of hydroelectrolytic disorders (Table 4).

### 3.7. Antidiabetic Therapy and Glycemia Levels

Our findings suggest that insulin is mainly used by autonomous and institutionalized patients (*p* = 0.0008, Pearson’s Chi-Square), while metformin (*p* = 0.003, Pearson’s Chi-Square) and GLP1 RA (*p* = 4 × 10^−6^, Fisher’s exact test) are mainly used by autonomous patients. On the other hand, Sulphonylureas are mostly used by male patients (*p* = 0.027, Pearson’s Chi-Square), while SGLT2 (*p* = 0.0004, Pearson’s Chi-Square) and GLP1 RA (*p* < 0.0001, Pearson’s Chi-Square) are mostly used by patients within the age group 65–85 years. Patients treated with sulfonylureas (*p* = 0.008), insulin and metformin association (*p* = 0.040), insulin and sulfonylurea association (*p* = 0.048), and DPP4i and sulfonylurea association (*p* = 0.031) were exposed to a higher risk of hyperglycemia (≥180 mg/dL) at admission (Table 5).

## 4. Discussion

### 4.1. Glycemia Levels and Patients’ Characteristics

Glycemic control in patients with T2DM is influenced by the type of family and social support they may have [27,28]. It is extremely important to include the patient, their family, and caretakers in the clinical decisions to achieve adherence to T2DM self-management, especially because much of the process takes place within the family environment [29]. Our findings suggest that patients with T2DM and good family support had better glycemic control upon admission to the emergency department compared to autonomous or institutionalized patients (Table 2). These results are in line with other studies in this field, according to which T2DM self-management education with family support improves health outcomes for patients with uncontrolled glycemia [30,31].

### 4.2. Impact of Hyperuricemia on Kidney Function and Cardiovascular System

HU is a condition that appears to be associated with increased insulin resistance and the onset and progression of diabetic complications [32]. Evidence provided by epidemiological studies suggests that HU is also a risk factor for HBP, CKD, and cardiovascular disease (CVD) [33]. HU in patients with T2DM is an important risk factor for both cardiovascular disease and kidney function deterioration [34,35,36,37]. An elevated uric acid level is a marker of cardiovascular risk. However, this association is not independent of some indicators of kidney function impairment, such as albuminuria or decreased glomerular filtration rate [38]. Several observational studies have identified HU as a risk factor for the development and/or progression of kidney disease, with some clinical trials suggesting that reducing uric acid levels with allopurinol could have beneficial effects in preventing or delaying kidney function deterioration [39]. However, recent studies have failed to statistically demonstrate the benefit of allopurinol on kidney function in these circumstances [40,41]. A likely explanation for the discrepancy between these studies and previous observational studies is that the predictive effect of HU on the loss of kidney function may be indirect and attributable to its association with other causally related features of kidney disease, such as insulin resistance and metabolic syndrome [42]. Regarding the potential effects of antidiabetic therapy in this domain, a recent study suggests that SGLT2i have an anti-inflammatory activity via uric acid and insulin by reducing the respective levels, with cardiovascular and renal benefits [43].

Our findings suggest that patients with HU had a higher incidence of acute cardiovascular disorders at admission. Moreover, patients with HU and higher glycemia levels (≥180 mg/dL) had higher levels of serum creatinine and urea and, therefore, greater worsening of kidney function, in line with what has been reported in other studies.

### 4.3. Relationship between Hemoglobin Levels, Gender, Age, and Kidney Function

Patients with T2DM are more susceptible to the development of chronic anemia, which may be due to inadequate glycemic control, CKD (that leads to erythropoietin production decrease), presence of T2DM complications, or age >60 years [44,45,46]. Previous studies have shown that diabetic females and diabetic elderly are the most vulnerable groups to anemia; moreover, having high blood creatinine is an important influence at this level [47,48]. According to our findings, female gender, older age, and serum creatinine values above 1.2 mg/dL, are related to lower hemoglobin levels.

### 4.4. Liver Function and Serum Glycemia Levels

Both AST and ALT have metabolic functions and are expressed in multiple organs, including the liver, myocardium, and skeletal muscle. ALT is mainly expressed in the liver, while AST is widely expressed, including in skeletal muscle. Therefore, a high serum ALT activity reflects the destruction of hepatocytes, while a high serum AST activity, along with a normal ALT activity, may reflect muscle damage [49,50]. Patients with T2DM have a higher incidence of liver functions tests showing abnormalities than individuals who do not have the disease. In addition, non-alcoholic fatty liver disease is very prevalent [51]. An increasing AST/ALT ratio is associated with declining glucose regulation, metabolic impairment, and organ dysfunction, including non-alcoholic fatty liver disease and cardiovascular disease [52]. Some recent studies reported that AST/ALT ratio increases is inversely related to metabolic syndrome development. It is accepted that an AST/ALT ratio is inversely associated with T2DM occurrence [53]. Our findings suggest that higher glycemia values at admission are associated with a lower AST/ALT ratio.

### 4.5. Patients’ Pharmacotherapeutic Profile and Acute Cardiovascular Disease

There is a close link between T2DM and cardiovascular diseases, which are the most prevalent cause of morbidity and mortality in diabetic patients [54,55]. Cardiovascular risk factors such as obesity, hypertension, and dyslipidemia are common in patients with T2DM, putting them at an increased risk for cardiovascular events [56,57]. T2DM is an important risk factor for the development of micro and macrovascular complications, including coronary artery disease, AKI, ACKD, and even stroke [58]. In patients with T2DM, glycated hemoglobin (HbA1c) level ≥ 7.0% was the strongest predictor of stroke and acute myocardial infarction [59]. Epidemiological studies have shown that a 1% increase in HbA1c levels leads to a 15 to 18% increase in cardiovascular events in patients with T2DM [60]. Intensive glycemic control aims to obtain an HbA1C of less than 7%, contributing to the prevention or the progression delay of microvascular complications, such as diabetic retinopathy and CKD, in patients with T2DM [24]. In contrast, the available information on the association between glycemic control and macrovascular diseases is limited since the glycemic control profiles of antidiabetic drugs in various cardiovascular complications have not yet been clearly elucidated [61,62]. Our findings indicate that obese patients, with or without concomitant dyslipidemia, showed a higher incidence of cardiovascular adverse events.

The recent realization that several antidiabetic drug classes approved may have divergent effects on HF and that some classes of agents may reduce the risk of HF has triggered different studies to establish a more predictable relationship between treatments and outcomes [63,64]. Some cardiovascular controlled trials have demonstrated the ability of iSGLT2 to reduce major cardiovascular adverse events and hospitalization for heart failure [65,66,67]. Dapagliflozin was even recently approved in Europe for the treatment of HF with reduced ejection fraction [68]. A recent study showed that metformin utilization compared to sulfonylureas in T2DM patients with worsening renal function was associated with reduced hospitalization for HF [69]. Although, recent large randomized controlled trials have not shown differences in cardiovascular risk of sulfonylureas versus pioglitazone or linagliptin [70]. Some studies point to a higher incidence of DHF in patients with T2DM taking sulfonylureas; others show inconclusive data [71].

Our findings demonstrated that patients using sulfonylureas, metformin, and iDPP4 association and metformin and sulfonylurea association were exposed to a lower risk of DHF. On the other hand, our findings did not show any benefit of SGLTi in this parameter (Table 3).

### 4.6. Patients’ Pharmacotherapeutic Profile and Kidney Disease

The pathophysiology of kidney disease in T2DM is characterized by multifactorial critical impairment. Hyperglycemic states lead to dysregulated intracellular metabolism, inflammatory kidney damage, increased apoptosis processes, and tissue fibrosis [72]. Higher HbA1c was associated with AKI in adults with T2DM and CKD, suggesting that improving glycemic control may reduce the risk of AKI [73]. The impact of antidiabetic drugs on renal function is increasingly studied. Dapagliflozin (SGLTi), for example, has been shown to be able to reduce the incidence of renal events and to prolong CKD patients’ survival, with and without T2DM [74].

Our findings demonstrated that patients undergoing treatment with metformin or metformin and DPP4i association were exposed to a lower risk of AKI or ACKD, revealing a protective effect (Table 4). Current experimental and clinical data provide some evidence for metformin as a promising pharmacological tool for renal diseases with or without T2DM [75]. Based on experimental evidence and some relevant clinical observations, metformin seems to be a promising drug in the treatment of progressive renal damage [76].

Antidiabetic drugs are associated with certain acid-base and HED, which should be taken into account in the decision process of T2DM treatment [77]. Our findings suggest that patients taking iSGLT2, insulin and sulfonylurea association, metformin and iSGLT2 association or iDPP4 and iSGLT2 were exposed to a higher risk of hydroelectrolytic disorders, unlike patients using ARGLP1, insulin, and iDPP4 association or iDPP4 and ARGLP1 association, that were exposed to a lower risk in this domain (Table 4). Although clinical data suggest that iSGLT2s are safe and protect against renal and cardiovascular events, little attention has been devoted to the effects of these compounds in the renal treatment of different electrolytes. Although a natriuretic effect and osmotic diuresis are expected, these compounds may also modulate the urinary excretion of potassium, magnesium, phosphate, and calcium. Some of the disturbances of homeostasis are transient, while others may persist, suggesting that the administration of these compounds may induce new electrolyte homeostasis [78].

### 4.7. Relationship between Patients’ Profile and Anti-Diabetic Drugs Used

According to our findings, metformin is more commonly used by autonomous patients (Table 5). This may arise because it is the recommended first-line treatment in T2DM and is used in earlier stages of the disease when most patients do not need any kind of support (familiar or institutional) [79]. Insulin is mostly used by autonomous and institutionalized patients because it is a therapeutic option that requires more controlled administration management [80]. GLP1 RA is mostly used by autonomous and younger patients. This fact may be due to its indication in obese patients and, therefore, in the reduction in the risks associated with this comorbidity. Furthermore, its subcutaneous administration also presupposes some autonomy or some type of support [81]. Sulphonylureas are used more by male than female patients. This comes in line with previous studies, according to which male gender and lower body mass indices are associated with a better glycemic response with sulfonylureas [82]. SGLT2i are mostly used by patients within the age group 65-85 years compared to older patients. In general, they are well tolerated; however, some caution is recommended in older patients who use other concomitant therapies such as diuretics [83].

### 4.8. Relationship between Glycemic Levels at Admission and Antidiabetic Drugs

The impact of antidiabetic drugs on glycemic control in the real world does not always match the results reported in clinical trials. In addition, non-adherence to therapy may be influenced by patient-centered and therapy-related factors [84,85,86,87]. According to some previous studies, the pattern of antidiabetic drugs’ use varies between different age groups and gender. Metformin, for example, due to its low risk for hypoglycemia, may be beneficial in older adults, and its low cost may make it an efficient choice [88,89]. On the other hand, factors related to therapy, including medication route, treatment duration, treatment complexity (a fixed-dose combination can lead to a significant improvement in adherence to pharmacological therapy of T2DM compared to a loose-dose combination), drug type, and drug side effects, are aspects that also influence the pattern of antidiabetic drugs’ use [90,91]. In addition, new-generation antidiabetic drugs are less studied than other, more traditional therapeutic options, so their effects, both in terms of efficacy and safety, may be less predictable, particularly in more vulnerable populations, such as the elderly [92]. Dosage adjustments are often required due to factors such as suboptimal medication adherence, psychosocial profiles, lifestyle, and health-seeking behavior [93,94]. Several comparative studies were carried out to assess the effectiveness of different antidiabetic drugs in glycemic control. In general, inadequately-controlled T2DM patients can benefit from using a combination of two or more different antidiabetic drugs [95,96]. If the HbA1C level is greater than 7.5% during treatment or if the baseline HbA1C is ≥9%, combination therapy with two oral antidiabetic drugs or with insulin may be considered [25,97]. There has been major progress in T2DM pharmacological treatment during the last few years. The rapid pace at which diabetology is developing makes it challenging to keep up with the interesting and innovative therapeutic approaches currently used [98].

Different recombinant insulin analogs have different mechanisms of action: rapid-acting insulin analogs provide a bolus level of insulin needed with meals (prandial insulin); longer-acting insulins released slowly over a longer period provide the required level of basal insulin throughout the day and night. Although insulin has been an important discovery for the treatment of DM, it is rarely used as a first-line treatment option for T2DM. Insulin administration carries risks of developing severe hypoglycemia and cardiovascular complications and occurs more often when patients develop insulin tolerance and an increase in doses is required [99,100].

Metformin monotherapy lowers fasting plasma glucose and glycated hemoglobin in a first-line approach to the T2DM patient. It is currently the only antihyperglycemic drug recommended by the American Diabetes Association and the European Association for the Study of Diabetes as initial oral therapy for patients with T2DM [101,102].

Sulfonylureas have been widely prescribed to treat T2DM. They are well tolerated, and their popularity can be attributed to their low cost and the possibility of being used as monotherapy or in combination with metformin [103]. Sulphonylureas interact not only with their receptors on pancreatic β-cells but also with smooth muscle cells and cardiac myocytes, which may explain why they are associated with a higher prevalence of hypoglycemia and cardiovascular risk [104,105]. However, most reports support the cardiovascular safety of sulfonylureas [106].

iDPP4s have been shown to be non-inferior to classical antidiabetic drugs and to be well tolerated with almost no side effects or episodes of hypoglycemia, with a neutral or slightly beneficial effect on body weight. Its oral administration and fixed doses without the need for escalation are other characteristics that facilitated adherence to this type of therapy. They are effective in improving glycemic control, mainly when used in association with metformin or other antidiabetic drugs, including insulin. The near absence of side effects and its weight neutrality, in contrast to sulfonylureas, thiazolidinediones, or even insulin, made this class of drugs one of the most used after metformin [107,108].

GLP-1 ARs promote glycemic control through a multitude of widely recognized physiological mechanisms, among them stimulation of insulin secretion and inhibition of glucagon release, directly improving postprandial glucose homeostasis, while inhibition of gastric emptying and food intake represents a long-term positive effect in limiting weight gain. Several cardiovascular outcomes studies have shown that GLP-1 RAs can effectively prevent cardiovascular events such as AMI or stroke and associated mortality. The underlying mechanisms may be related to the inhibition of the progression of atherosclerotic lesions. Therefore, the guidelines particularly recommend GLP-1 RA treatment in patients with pre-existing atherosclerotic vascular disease [109,110].

iSGLT2s binds competitively to glucose transporters reducing its reabsorption by renal tubular epithelial cells, promoting urinary excretion and, consequently, exerting hypoglycemic effects. They have demonstrated good effectiveness in glycemic control in a non-insulin-dependent way [111,112].

Our findings suggest that patients treated with insulin (when associated with metformin or sulfonylurea) and sulfonylureas (with or without DPP4i associated) were exposed to a higher risk of hyperglycemia at admission (Table 5). Some studies demonstrated that patients on insulin were more likely to have poorly controlled glycemia than those on metformin alone [113,114]. Sulphonylureas are generally associated with hypoglycemia risk, particularly in the elderly, which contradicts our findings [115]. Although, most of these patients presented clinical conditions of considerable stress. Stress-induced hyperglycemia is a condition that develops in patients undergoing any form of clinical stress and that occurs due to an increase in peripheral insulin resistance, a decrease in its secretion, and increased glucose production [116]. Sulphonylureas stimulate insulin secretion, metformin decreases glucose synthesis, and endogenous insulin acts on its specific receptors. All these mechanisms are affected by stress-induced hyperglycemia, and a more robust approach to glycemic control and stabilization may be necessary. The treatment consists of intravenous insulin infusion [117].

## 5. Conclusions

T2DM patients are characterized by great heterogeneity from a clinical point of view. Usually, they have several associated comorbidities, so the pharmacotherapeutic approach must consider all aspects that may affect the progress of the disease. Health professionals should contribute to improving patients’ health literacy, as it directly influences management and self-care skills, glycemic control, and patients’ quality of life, contributing to better health outcomes. 

Our findings corroborate some of the previously established relationships, both in clinical and pharmacological terms, as well as demonstrate that patients have better glycemic control and disease management when they benefit from adequate social and family support. However, some aspects may have influenced our results: this is a monocentric study that includes patients from a restricted geographic area with advanced mean age and several associated comorbidities; most patients have chronic pharmacotherapeutic plans that include different drugs with a wide range of effects and interactions; there is no information available on the stage of diabetic disease in which each patient; most of these patients were in a complex clinical situation at admission.

## Figures and Tables

**Table 1:** Patients’ characteristics at admission.

Age					
Mean	80.59	Standard Deviation	7.92	Min-Max	65–99
			Gender		
			Male (n = 204)		Female (n = 216)
Autonomous			94 (22.4%)		61 (14.5%)
Family support			49 (11.7%)		42 (10.0%)
Institutionalized			61 (14.5%)		113 (26.9%)
**Pathological history previous to admission**					
Type 2 Diabetes Mellitus			204 (48.6%)		216 (51.4%)
High blood pressure			173 (41.2%)		181 (43.1%)
Heart failure			77 (18.3%)		98 (23.3%)
Atrial fibrillation			38 (9.0%)		45 (10.7%)
Acute myocardial infarction			14 (3.3%)		17 (4.0%)
Dyslipidemia			82 (19.5%)		98 (23.3%)
Chronic kidney disease			65 (15.5%)		91 (21.7%)
Hyperuricemia			30 (7.1%)		42 (10.0%)
Stroke			35 (8.3%)		39 (9.3%)
Obesity			27 (6.4%)		44 (10.5%)
Chronic liver disease			17 (4.0%)		6 (1.4%)
Oncological disease			25 (6.0%)		11 (2.6%)
Chronic obstructive pulmonary disease			19 (4.5%)		17 (4.0%)
**Diagnosis at admission**					
Decompensated heart failure			43 (10.2%)		66 (15.7%)
Acute chronic kidney disease			46 (11.0%)		67 (16.0%)
Acute kidney injury			66 (15.7%)		51 (12.1%)
Urinary tract infection			14 (3.3%)		28 (6.7%)
Pulmonary embolism			12 (2.9%)		9 (2.1%)
Stroke			18 (4.3%)		19 (4.5%)
Acute myocardial infarction			9 (2.1%)		8 (1.9%)
Respiratory tract infection			56 (12.9%)		65 (15.5%)
Hydroelectrolytic disorders			94 (22.4%)		92 (21.9%)
Bleeding			3 (0.7%)		0 (0.0%)
Gastroenteritis			4 (1.0%)		2 (0.5%)
Acute chronic liver disease			12 (2.9%)		5 (1.2%)
Pancreatitis			3 (0.7%)		3 (0.7%)
Hypoglycemia			2 (0.5%)		2 (0.5%)
Respiratory failure			59 (14.0%)		74 (17.6%)
Sepsis			5 (1.2%)		11 (2.6%)
**Antidiabetic drugs included in the therapeutic plan before admission**					
Insulin			100 (23.8%)		127 (30.2%)
Sodium-glucose cotransporter inhibitors			54 (12.9%)		54 (12.9%)
Dipeptidyl peptidase inhibitors			111 (26.4%)		107 (25.5%)
Glucagon-like peptide-1 receptor agonists			10 (2.4%)		17 (4.0%)
Metformin			107 (25.5%)		96 (22.9%)
Sulfonylureas			36 (8.6%)		22 (5.2%)
**Laboratory parameters at admission**					
Glycemia			Blood creatinine		
**<180 mg/dL**		≥180 mg/dL	<1.2 mg/dL		≥1.2 mg/dL
183 (43.6%)		237 (56.4%)	170 (40.5%)		250 (59.5%)

**Table 2:** Relationship between patient’s condition, gender, and age with glycemia levels.

Condition					
Glycemia	Autonomous	Family Support	Institutionalized	Total	*p*-Value
<180 mg/dL	58 (13.8%)	51 (12.1%)	74 (7.6%)	183 (43.6%)	0.016 *
≥180 mg/dL	97 (23.1%)	40 (9.5%)	100 (23.8%)	237 (56.4%)	
Total	155 (36.9%)	91 (21.7%)	174 (41.4%)	420 (100%)	

* Pearson’s Chi-Square.

**Table 3:** Relationship between decompensated heart failure and antidiabetic therapy.

Previously Diagnosed Heart Failure			Decompensated Heart Failure at Admission		
Yes	No		Yes	No	
175 (41.7%)	245 (58.3%)		109 (26.0%)	311 (74.0%)	
Antidiabetic Drugs	Decompensated HF (No/Yes)		*p*-Value	OR	Confidence Interval 95%
Insulin	155 (49.8%)	72 (66.1%)	*p* = 0.003 *	1.959	1.243–3.085
GLP1 RA	15 (4.8%)	12 (11.0%)	*p* = 0.023 *	2.441	1.105–5.395
Sulfonylureas	51 (16.4%)	7 (6.4%)	*p* = 0.009 *	0.350	0.154–0.796
Insulin + GLP1 RA	9 (2.9%)	10 (9.2%)	*p* = 0.007 *	3.389	1.339–8.579
Metformin + DPP4i	100 (32.2%)	23 (21.1%)	*p* = 0.029 *	0.564	0.336–0.947
Metformin + Sulfonylurea	38 (12.2%)	3 (2.8%)	*p* = 0.003 **	0.203	0.061–0.673

* Pearson’s Chi-Square. ** Fisher’s exact test.

**Table 4:** Relationship between antidiabetic therapy and acute kidney injury, acute chronic kidney injury, and hydroelectrolytic disorders.

Antidiabetic Drugs	AKI or ACKD (n = 230)	*p*-Value	OR	Confidence Interval 95%
Metformin	99 (43.0%)	0.017 *	0.625	0.424–0.920
Metformin + DPP4i	56 (24.3%)	0.014 *	0.521	0.387–0.902
	Hydroelectrolytic disorders			
SGLT2i	64 (34.4%)	0.0003 *	2.265	1.450–3.539
GLP1 RA	6 (3.2%)	0.017 *	0.338	0.134–0.856
Insulin + DPP4i	38 (20.4%)	0.034 *	0.614	0.390–0.967
Insulin + Sulfonylurea	8 (4.3%)	0.026 **	5.213	1.094–24.853
Metformin + SGLT2i	34 (18.3%)	0.026 *	1.870	1.071–3.264
DPP4i + SGLT2i	29 (15.6%)	0.007 *	2.358	1.252–4.440
DPP4i + GLP1 RA	0 (0.0%)	0.003 **	0.957	0.932–0.984

* Pearson’s Chi-Square. ** Fisher’s exact test.

**Table 5:** Relationship between Antidiabetic drugs and serum glycemia level at admission.

Antidiabetic Drugs	Glycemia ≥ 180 mg/dL (No/Yes)		*p*-Value	OR	Confidence Interval 95%
Sulfonylureas	16 (8.7%)	42 (17.7%)	*p* = 0.008 *	2.248	1.219–4.145
Insulin + Metformin	20 (10.9%)	43 (18.1%)	*p* = 0.040 *	1.806	1.022–3.194
Insulin + Sulfonylurea	1 (0.5%)	9 (3.8%)	*p* = 0.048 **	7.184	0.902–57.227
DPP4i + Sulfonylurea	11 (6.0%)	29 (12.2%)	*p* = 0.031 *	4.644	1.058–4.492

* Pearson’s Chi-Square. ** Fisher’s exact test.

## Data Availability

Not applicable.
